# Thymic function recovery after unrelated donor cord blood or T-cell depleted HLA-haploidentical stem cell transplantation correlates with leukemia relapse

**DOI:** 10.3389/fimmu.2013.00054

**Published:** 2013-03-04

**Authors:** Emmanuel Clave, Daniela Lisini, Corinne Douay, Giovanna Giorgiani, Marc Busson, Marco Zecca, Francesca Moretta, Gloria Acquafredda, Letizia P. Brescia, Franco Locatelli, Antoine Toubert

**Affiliations:** ^1^Departement d'Immunologie, INSERM UMRS-940, AP-HPParis, France; ^2^Université Paris Diderot, Sorbonne Paris CitéParis, France; ^3^Pediatric Hematology-Oncology, IRCCS Policlinico San Matteo FoundationPavia, Italy; ^4^Department of Pediatric Hematology and Oncology, University of Pavia, IRCCS Ospedale Bambino GesùRome, Italy; ^5^Hôpital St-Louis, AP-HPParis, France

**Keywords:** HSCT, thymic function, T cells, relapse, leukemia

## Abstract

Use of alternative donors/sources of hematopoietic stem cells (HSC), such as cord blood (CB) or HLA-haploidentical (Haplo)-related donors, is associated with a significant delay in immune reconstitution after transplantation. Long-term T-cell immune reconstitution largely relies on the generation of new T cells in the recipient thymus, which can be evaluated through signal joint (sj) and beta T-cell-Receptor Excision Circles (TREC) quantification. We studied two groups of 33 and 24 children receiving, respectively, HSC Transplantation (HSCT) from an HLA-haploidentical family donor or an unrelated CB donor, for both malignant (46) and non-malignant disorders (11). Relative and absolute sj and beta-TREC values indicated comparable thymic function reconstitution at 3 and 6 months after the allograft in both groups. Compared to children with non-malignant disorders, those with hematological malignancies had significantly lower pre-transplantation TREC counts. Patients who relapsed after HSCT had a significantly less efficient thymic function both before and 6 months after HSCT with especially low beta-TREC values, this finding suggesting an impact of early intra-thymic T-cell differentiation on the occurrence of leukemia relapse.

## Introduction

Whenever an HLA-identical related donor is not available, alternative sources of donors/stem cells are to be used for Allogeneic Hematopoietic Stem Cell Transplantation (HSCT) (Beatty et al., 1995). Both unrelated Cord Blood (CB) and HLA-haploidentical (Haplo) family donors provide an effective treatment for many malignant and non-malignant diseases in children (Rocha and Locatelli, 2008). For the latter, the HLA disparity in the donor/recipient pair may lead to a beneficial and selective graft-versus-leukemia (GVL) effect, associated with natural killer (NK)-cell mediated alloreactivity (Ruggeri et al., 2002). Despite improvement in the graft procedure such as use of T-cell depletion (TCD) and infusion of high doses of CD34+ cells, which allows engraftment and reduces graft-versus-host disease (GvHD) in Haplo-HSCT recipients (Aversa et al., 1994; Handgretinger et al., 2001), a significant delay in immune reconstitution still remains a relevant problem, leading to a high incidence of both opportunistic infection and, in the absence of NK alloreactivity, to relapse (Ball et al., 2005; Wils et al., 2011). In this respect, association between poor clinical outcome and low lymphocyte counts early after CB- or Haplo-HSCT (Ciurea et al., 2011) or at month 6 in case of T-cell depleted HSCT (Novitzky et al., 2002) has been described. Moreover, we showed recently, in 33 Haplo-HSCT patients an association between low thymic function and an increased risk of leukemia relapse (Clave et al., 2012).

Recently, tools have been developed to monitor thymic function. Signal-joint T-cell-Receptor (TCR) Excision Circles (sjTREC) are small episomal DNA that results from the deletion of the TCR δ region during TCR α locus rearrangement. They are not replicated during lymphocyte cell division and reflect, in the periphery, the number of new lymphocytes generated in the thymus (Douek et al., 1998; Dion et al., 2007). BetaTREC are produced during TCR Dβ–Jβ recombination, occurring before TCR α-chain recombination in αβ T-cell differentiation. BetaTREC counts may therefore reflect the early stages of intrathymic differentiation and sj/beta TREC ratios the T-cell proliferation rate between β- and α-chain recombination events. Quantification of sj and beta-TREC molecules in peripheral blood cells has provided useful information in studies addressing the issues of aging, HIV infection, and HSCT (Dion et al., 2004, 2007), but have not yet been used in the context of studies on leukemia relapse after HSCT.

In view of these considerations, we decided to investigate thymic function recovery in patients given unrelated donor CB transplantation (UCBT) and to compare the results obtained in this cohort of patients with those of children given Haplo-HSCT. In detail, we retrospectively evaluated thymic function recovery through sj and beta TREC quantification in 57 pediatric patients, 33 receiving Haplo-HSCT previously reported and 24 given UCBT. Thymic function recovery was found to be similar in both groups. Moreover, correlation of TREC values with clinical parameters revealed a link between recovery of thymic function and specific disease. Indeed, we found that pre-transplantation sj and beta TREC were lower for patients with malignant disease. Moreover, there was a strong correlation between low thymic function persisting at 6 month after HSCT and a higher incidence of relapse in the Haplo-HSCT group.

## Patients

Two groups of pediatric patients, 24 receiving UCBT and 33 given Haplo-HSCT (Table 1) for either malignant or non-malignant diseases were studied, the last group being the same reported in our previously published study (Clave et al., 2012). In order to be included, patients had to be alive 6 months after transplantation (so, 3 patients that relapsed before 6 months were also included). All patients had been transplanted between October 2004 and February 2007 after having received a fully-myeloablative conditioning regimen. Details on patient and donor characteristics, as well as on transplantation outcome, are reported in Table 1. No patient given Haplo-HSCT received post-transplantation pharmacologic immune suppression, while those transplanted with CB cells received a combination of CsA and steroids (see also Table 1 for details). For the purpose of the study, patients with acute leukemia transplanted in 1st complete remission (CR) or in 2nd CR after a relapse occurring more than 6 months after treatment discontinuation, as well as patients affected by refractory cytopenia, were assigned to the early disease group. All other patients were included in the advanced disease group. Acute and chronic GvHD (cGvHD) were diagnosed and graded according to the Seattle criteria (Glucksberg et al., 1974; Storb et al., 1983). Patients surviving more than 14 and 100 days post-transplantation were evaluated for acute and cGvHD, respectively. The study was approved by the Ethical committee of Policlinico San Matteo, Pavia, Italy (approval number 446/DG).

**Table 1:** **Patient-, donor-characteristics, and transplant outcomes**.

	**Haplo-HSCT *N* = 33**	**UCBT *N* = 24**	***p***
**RECIPIENT**			
Male, *N* (%)	23 (70%)	14 (58%)	0.411
Median age, years (range)	7.7 (3–17)	4.7 (1–16)	<0.001
*Hematological malignancies*	27 (82%)	19 (79%)	0.999
Acute lymphoblastic leukemia	20	11	
Acute myeloid leukemia	3	6	
Myelodysplastic syndromes	3	0	
Juvenile myelomonocytic leukemia	1	2	
*Other diagnosis*			
Hemophagocytic lymphohistiocytosis	0	5	
Fanconi anemia	3	0	
Congenital amegakaryocytic thrombocytopenia	2	0	
Blackfan-Diamond anemia	1	0	
**TRANSPLANTATION**			
*Source of cells* PB/CB	33/0	0/24	<0.001
*Conditioning regimen*^a^: TBI/chemo-based	24/9	9/15	0.014
*GvHD prophylaxis*^b^: TCD/CsA + steroids	33/0	0/24	<0.001
*Infused CD34+ cells*: Median (range) × 10^6^/Kg	22 (8.7–41)		
*Infused nucleated cells*: Median (range) × 10^7^/Kg		5.05 (1.4–12.5)	
**CLINICAL OUTCOMES**			
*Acute GvHD*			
Grade (I/II/III/IV)	6/4/1/0	4/8/1/0	
Grade II–IV	5 (15%)	9 (37%)	0.067
*Chronic GvHD*	6 (18%)	2 (8%)	0.446
*Relapse*	8 (24%)	3 (12%)	0.326
*Serious infections*^c^	20 (61%)	13 (54%)	0.786

Haplo-HSCT indicates HLA-Haploidentical Hematopoietic Stem Cell Transplantation; UCBT, Unrelated Cord Blood Transplantation; CsA, cyclosporin A; GvHD, graft-versus-host disease; PB, peripheral blood; TBI, total body irradiation.

a TBI-based conditioning regimen was employed in 33 children and consisted of: fractionated TBI (12 Gy over 6 fractions in 3 days), Thiotepa (10 mg/Kg in 2 doses) and fludarabine (160 mg/m^2^ over 4 days). Chemotherapy-based conditioning regimen were as follows: 12 patients received Busulfan (16 mg/Kg in 16 doses over 4 days), Cyclophosphamide (120 mg/Kg in 2 days) and melphalan (140 mg/m^2^ in single dose); 7 children received Busulfan (16 mg/Kg in 16 doses over 4 days), Thiotepa (10 mg/Kg in 2 doses) and fludarabine (160 mg/m^2^ over 4 days); and 5 children were given Treosulfan (14 gr/m^2^ for 3 consecutive days), Thiotepa (10 mg/Kg in 2 doses) and fludarabine (160 mg/m^2^ over 4 days).

b Patients receiving Haplo-HSCT were transplanted with CD34+ selected cells and were not given any immune-suppressive drug after transplantation. Patients transplanted with cord blood cells received a combination of Cyclosporine-A (3 mg/Kg/day) and steroids [methylprednisolone (2 mg/Kg/day)] as GvHD prophylaxis.

c Reactivation of viral infections (i.e., cytomegalovirus and Epstein-Barr virus) and proven/probable invasive aspergillosis.

## Methods

### Design of the study

DNA samples of patients enrolled in the study were collected before transplantation and at 3 and 6 months after the allograft. At the same time-points, we evaluated absolute number and percentage of the different T lymphocyte subsets.

### sjTREC and beta-TREC quantification

Genomic DNA was extracted from Peripheral Blood Mononuclear Cells (PBMC) using the QIAamp DNA Blood Minikit (Qiagen, Hilden, Germany). Quantification of sjTREC and beta-TREC was performed by real-time quantitative PCR (qPCR) (ABIPRISM7500, Applied Biosystems, Foster City, CA), as previously described (Clave et al., 2009). Briefly, a first PCR reaction was carried out in multiplex with 3 different outer primer mixes, 1–5 μg of genomic DNA, 200 μM each dNTP, 2.5 mM MgCl2, 1× buffer and 1.25 unit of *Platinum*® Taq polymerase (Invitrogen, Cergy-Pontoise, France) in 50 μL (10 min at 95°C, then 19 cycles of 95°C, 30 s; 60°C, 30 s and 72°C, 2 min). Final quantification was made on ABI PRISM 7700, in duplicate with a second multiplex reaction that contains 5 μL of a 1/100 or 1/1000 dilution of the first PCR product, inner primers, and probe for sjTREC or one of the Dβ–Jβ segments, inner primers and probe for albumin (alb) gene, 1.25 mM each dNTP, 3 mM MgCl2, 1× buffer and 1.25 unit of *Platinum*® Taq polymerase in 25 μL (5 min at 95°C then 40 cycles of 95°C, 15 s and 60°C, 1 min). The sum of the 10 Dβ–Jβ segments was finally multiplied by 1.3 to extrapolate for all the 13 existing Dβ–Jβ segments. All primers and probes have been obtained from Eurogentec (Seraing, Belgium) except the alb vic-labeled probe from Applied Biosystems.

TREC data were validated only if at least 50,000 genome equivalents were detected by alb qPCR; therefore, some time-points were missing for 5 patients but other negative TREC values were not due to absence of genomic DNA. Data were first expressed per 150,000 PBMC and the total number of TREC per μL of blood was calculated using the absolute white blood cells count at time of sample collection.

### Flow cytometry analysis

FITC, PE, PerCP, or APC monoclonal antibodies (MoAbs) specific for the following antigens were employed for the evaluation of lymphocyte subsets: CD45, CD3, CD4, CD8 (BD Biosciences, Mountain View, CA). Appropriate isotype-matched controls (BD Bioscience) were included. Three-color or four-color cytometry, through direct immune fluorescence and FACSCalibur or FACSCanto cytometer (BD Biosciences), was performed.

### Statistical analysis

Non-parametric Mann–Whitney or Kruskal–Wallis tests were used to correlate the effect of different clinical parameters on TREC counts. Since data did not have a normal distribution, a logarithmic transformation was performed. Difference in cumulative incidence of relapse was estimated using univariate Kaplan–Meier analysis. *P*-values <0.05 were considered to be statistically significant. All analyses were performed using SPSS Statistics 20 Software.

## Results

### Thymic function recovery is similar after haplo-HSCT and UCBT in pediatric patients

The distribution of circulating CD3^+^, CD4^+^, or CD8^+^ T-lymphocyte subsets, 3 and 6 months after HSCT, was variable, especially for patients given Haplo-HSCT (Table 2). At month 3, CD3+, CD4+, and CD8+ cell counts/μL blood were lower in Haplo-HSCT than in UCBT patients (*p* = 0.003, 0.005, and 0.010, respectively). At month 6, only CD4+ cells remained significantly lower (*p* = 0.022) in the former group. These results confirm the detrimental role of TCD of the graft on early reconstitution of mature T cells and the role played by homeostatic expansion of T cells transferred with the graft in the case of UCBT (Roux et al., 1996).

**Table 2:** **Median number (range) of circulating T-lymphocyte subsets three (M3) and six (M6) months after HSCT (cells × 10^3^/μl)**.

		**M3**				**M6**			
		***N***	**CD3+**	**CD4+**	**CD8+**	***N***	**CD3+**	**CD4+**	**CD8+**
All patients	Haplo	33	264 (0–2666)	68 (0–1767)	80 (0–1666)	30	610 (140–2992)	242 (88–760)	270 (14–2596)
	UCBT	23	540 (180–4661)	190 (72–885)	280 (18–4092)	21	852 (352–6440)	336 (105–1288)	456 (80–5152)
	*p*		0.003	0.005	0.010		0.058	0.022	0.151
Malignant Haplo-HSCT	Relapse	8	279 (4–572)	29 (0–264)	84 (0–308)	7	867 (154–2460)	234 (98–369)	480 (14–2132)
	CR	19	252 (0–2666)	138 (0–1767)	63 (0–1666)	17	560 (140–2992)	273 (88–546)	248 (14–2596)
	*p*		0.894	0.300	0.831		0.193	0.975	0.075
Malignant UCBT	Relapse	3	1040 (266–4661)	234 (91–885)	819 (154–3540)	3	1250 (852–1288)	324 (250–529)	759 (516–1000)
	CR	15	715 (180–4465)	153 (72–616)	476 (18–4092)	14	811 (352–6440)	322 (105–1288)	442 (80–5152)
	*p*		0.441	0.594	0.441		0.314	0.801	0.313

M3 stands for Month 3; M6, Month 6, and CR, Complete remission.

**Figure 1:**
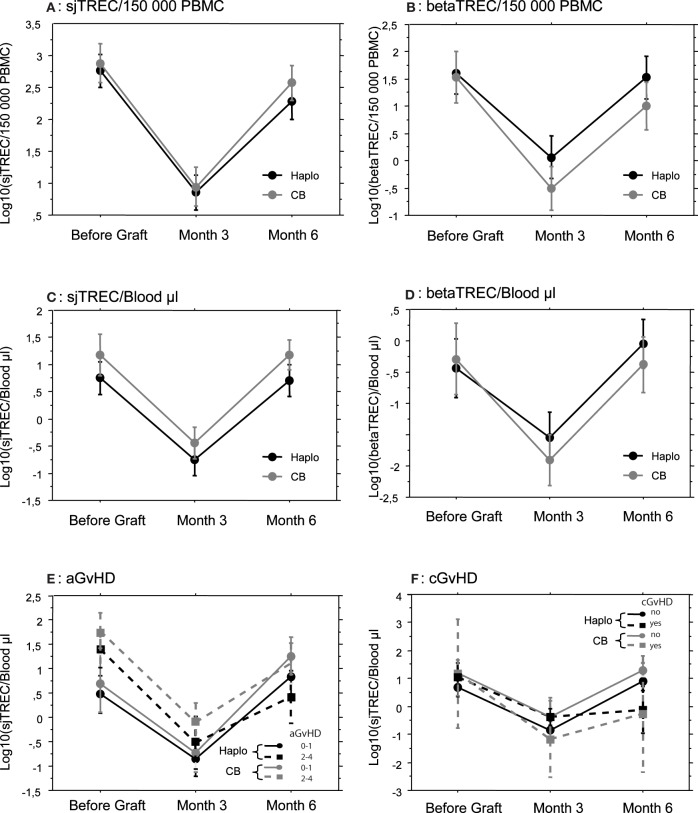
**Thymic reconstitution is not dependent on the stem cell source employed or GvHD occurrence.** Mean (±SE) number of log10 TREC was measured by quantitative PCR, before, and 3 and 6 months after transplantation in patients that received either a haploidentical hematopoietic Stem Cell Transplantation (Haplo, *N* = 33) or a Cord Blood Unrelated Donor Graft (CB, *N* = 24). Signal Joint (sj) TREC were quantified in (**A,C,E,** and **F**) and betaTREC in (**B** and **D**). Results were expressed by 150,000 Peripheral Blood Mononuclear Cells in (**A** and **B**) and by μL of blood in (**C,D,E,** and **F**). Patients were subsequently subdivided according to GvHD occurrence (**E** and **F**).

Reconstitution of newly generated naïve T cells relies on thymic differentiation of donor-derived lymphoid progenitors. We, therefore, evaluated recovery of thymic function through the quantification of sjTREC and beta-TREC on the samples collected at the same points in which lymphocyte counts were investigated. Before the allograft, the median number of sjTREC per 150,000 PBMC were 1375 (range 0–27,612) and 2035 (range 0–16,626) for Haplo-HSCT and UCBT patients, respectively (*p* = not significant, NS). Three months after the allograft the median number of sjTREC per 150,000 PBMC had dropped to 27 (0–1973) and 45 (0–1284) for Haplo-HSCT and UCBT patients, respectively. At 6 months, although there was a great inter-patient variability, the median number of sjTREC numbers almost returned to pre-graft levels with 281 (0–31,286) and 647 (0–16,395) sjTREC/150,000 PBMC, for Haplo-HSCT and UCBT patients (Figure 1A), respectively. The recovery of betaTREC paralleled that of sjTREC (Figure 1B) without any significant difference between the two groups of patients. Accordingly, at each time points, the number of sjTREC highly correlated with the number of beta-TREC (*r* = 0.66, *p* < 0.001, Pearson correlation).

Since proliferation of memory or effector T cells could affect TREC number per PBMC by dilution, we also expressed sjTREC and beta-TREC counts per microliter of patient blood using absolute cell counts. Absolute numbers of sjTREC (Figure 1C) and beta-TREC (Figure 1D) followed the same kinetics as numbers of TREC/150,000 PBMC, without difference between Haplo-HSCT and UCBT patients, this ruling out an indirect, confounding effect of T-cell proliferation. Only absolute sj and beta-TREC numbers per μL will be used afterwards.

Since sjTREC value as a marker of thymic output is dependent on age (Douek et al., 1998), we also compared the median age of patients in the two groups. Haplo-HSCT patients were indeed significantly older (*p* = 0.0008) than UCBT patients (median age being 7.7 and 4.7 years, range 3–17 and 0.75–16 years, respectively). Thus, from the observed TREC values, we can conclude that, despite an older age, thymic reconstitution was equally effective in patients given either Haplo-HSCT or UCBT.

Since GvHD has a major impact on immune reconstitution after allogeneic HSCT, we studied its influence on both groups of patients. There were less cases of acute GvHD (aGvHD) in Haplo-HSCT patients (Table 1). We did not see any significant difference for sjTREC/μL of blood between patients without or with grade I aGvHD and patients with grade II and III aGvHD (Figure 1E), in both groups of patients. Incidence of cGvHD was low in both groups with 6 (18%) and 2 cases (8%) in the Haplo-HSCT and UCBT cohorts, respectively. TREC values were lower at month 6, in both groups, when cGvHD occurred, but the difference was not statistically significant (Figure 1F).

### Patients affected by hematological malignancies have lower pre-transplant thymic function

**Figure 2:**
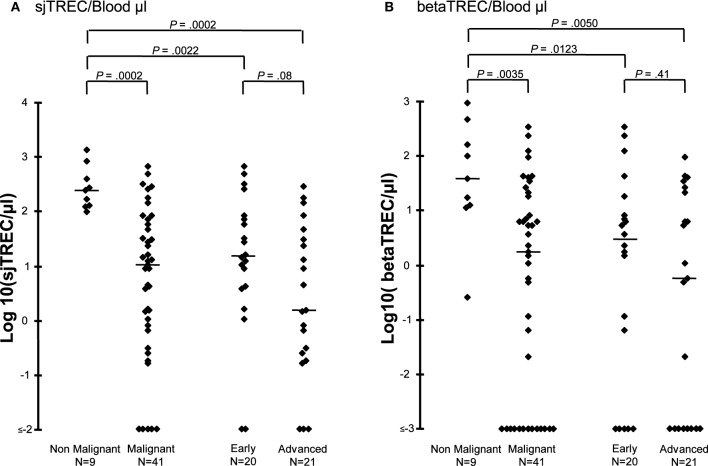
**Thymic function before transplantation is lower in patients affected by a hematological malignancy.** Log10 sjTREC/blood μL **(A)** and Log10 betaTREC/blood μL **(B)** are shown before HSCT for patients diagnosed with either a malignant or a non-malignant hematological disease. Patients with malignancy were subsequently subdivided according to their disease status. Horizontal bars represent the median.

Diagnosis may also have an impact on recovery of thymic function (Petridou et al., 2002; Clave et al., 2005). Indeed, TREC values before transplantation were significantly lower in the 46 patients with hematological malignancies (median 10 sjTREC/μL blood, range 0–674) than in the 11 with non-malignant disorders (median, 235 sjTREC/μL blood, range 97–1340, *p* = 0.0002) (Figure 2). This finding was not influenced by the type of allograft that the patients received (data not shown). There was no significant difference in age between the two groups of patients, affected or not by hematological malignancy (median 6.6 vs. 5.0 years, range 1–9 vs. 1–17, *p* = 0.18) that could explain a lower thymic function in patients with malignancy. Before transplantation, patients with early disease had higher sj and beta-TREC than those with more advanced disease (Figure 2). Moreover, median numbers of betaTREC/μL of blood were also significantly lower in patients treated for malignancies (median 1.7 vs. 39, *p* = 0.004) (Figure 2), suggesting an effect of the disease itself or of previous treatment on the T-cell progenitor compartment or before β-chain recombination.

### A low thymic function is associated with a higher relapse risk

We have previously shown in the group of Haplo-HSCT patients that a low thymic function before transplantation or after 6 months, was associated with an increased risk of relapse (Clave et al., 2012). Here, thymic reconstitution appears similar after both UCBT and Haplo-HSCT. Thus, we decided to merge the 19 UCBT to the 27 Haplo-HSCT cases affected by malignancies. Among the 46 patients treated for malignancies, 11 relapsed: 8 after Haplo-HSCT and 3 after UCBT (see Table 1). The median time between transplantation and relapse was 10 months (range 1.6–18 months).

**Figure 3:**
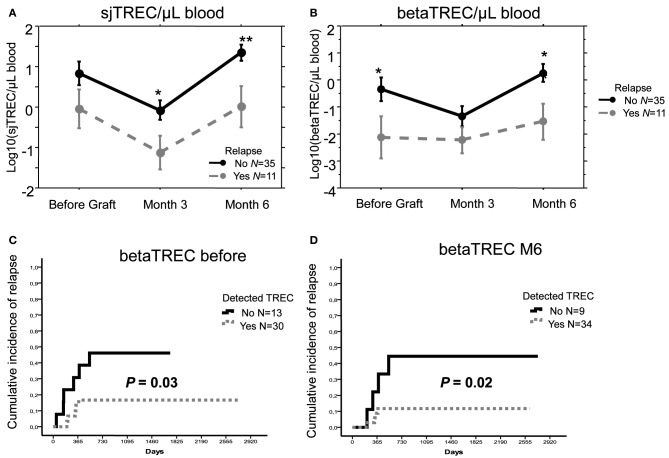
**A low thymic function is associated with a higher relapse risk.** Mean (±SE) number of log10 sjTREC/ Blood μL **(A)** and betaTREC/ Blood μL **(B)**, before, 3 and 6 months after transplantation in patients with hematological malignancies that received either a T-cell depleted haploidentical Hematopoietic Stem Cell Transplantation or an Unrelated Donor Cord Blood Transplantation (Mal.) or the former type of allograft only (Haplo). Patients who experienced relapse were represented with gray dotted lines, while patients maintaining complete remission with black plain lines. ^*^p < 0.05, ^**^p < 0.01. Cumulative incidence of relapse in patients affected by malignant disorders who did or did not have detectable betaTREC before **(C)**, and at month 6 after graft **(D)** (*p* in Log Rank test).

When considering both patients given UCBT and those receiving Haplo-HSCT, the median number of sjTREC/μL blood was low at 3 months and returned to the initial level at month 6. With a median follow-up of 43 months, patients who relapsed had lower values than the relapse-free patients both before transplantation (*p* < 0.05) and during follow-up (*p* = 0.014 and 0.006, at 3 and 6 months, respectively) (Figure 3A). Values of beta-TREC before transplantation and, even more significantly, after 6 months (Figure 3B) were also lower in patients who subsequently relapsed (*p* = 0.037 and 0.009, respectively).

We also carefully evaluated the occurrence of relapse in patients with a poor thymic function. We calculated the cumulative incidence of relapse in patients with a detectable thymic function compared to those without any thymic function (based on the detection sensitivity of the sj and beta TREC assays less than 0.1 and 0.01/150,000 PBMC, respectively). Because values at month 3 were too low to define this cut-off reliably, we studied only the values before transplantation and those at month 6 post transplantation. Absence of detectable sjTREC at each time point correlated with an increased incidence of relapse, although this correlation was not statistically significant, due to the limited number of events (data not shown). More notably, lack of detectable betaTREC before and at month 6 after the allograft was strongly associated to a higher incidence of relapse (*p* = 0.03 and 0.02, respectively) (Figures 3C,D). Use of median sj or beta-TREC value to split patient groups as low or high TREC gave similar results (data not shown). We also analyzed, in patients treated for malignancies, other parameters that have been shown to influence relapse rate, but neither age (*p* = 0.28), high-risk disease (*p* = 0.73), conditioning regimen (*p* = 0.24) nor number of CD34+ cells infused (for Haplo-HSCT patients, *p* = 0.72) had a significant impact on relapse in univariate analysis in this relatively small cohort of patients.

## Discussion

Alternative sources of stem cells/donors are increasingly used in the treatment of children with both malignant and non-malignant diseases in need of an allograft (Copelan, 2006). However, their use remains associated with delayed immune reconstitution and a high rate of infectious complications (Aversa et al., 1998). Using sj and beta-TREC quantification, we found that thymic function had the same pattern of reconstitution in patients that had been transplanted from either an HLA haploidentical family donor or an unrelated CB donor despite the difference in stem cell source and transplant procedure. It is known that TCD of the graft (*in vivo* or *ex vivo*) has a huge impact on early reconstitution of the lymphocyte compartment, since patients cannot benefit from the homeostatic expansion of lymphocytes transferred with the graft (Roux et al., 1996). Accordingly, we found that reconstitution of T-cell subset numbers (i.e., CD4+ and CD8+ T cell subpopulations) was faster in UCBT patients than in those given Haplo-HSCT. However, median values of beta and sjTREC were similar in both groups, this indicating that, despite TCD of the graft, a larger use of total body irradiation (Table 1) and an older age for Haplo-HSCT recipients, recovery of thymic function was equally effective in patients given either Haplo-HSCT or UCBT. These results also illustrate that the TREC assay provides additional and more detailed information on T-cell reconstitution, in particular on that of newly re-generated T lymphocytes which mainly accounts for the medium and long-term patient's immune competence, than crude T cell subsets counts.

We found a very limited impact of GvHD (Hazenberg et al., 2002; Storek et al., 2002; Clave et al., 2009), and especially of aGvHD, on recovery of thymic function in both groups. This could be explained by both the limited incidence of this complication in the 2 groups and by the young age of our patients. Indeed, in a previous study we reported that young age at time of the allograft correlates with a better thymic function recovery (Clave et al., 2009).

In accordance with what we and others have previously shown (Petridou et al., 2002; Chen et al., 2005; Clave et al., 2005), patients with malignant diseases had lower TREC values already before the allograft than those with non-malignant disorders. However, in those previously published studies, the groups were not matched for age (Petridou et al., 2002; Clave et al., 2005) and only the relative number of TREC was assessed (Petridou et al., 2002; Chen et al., 2005), leaving the possibility of TREC dilution due to peripheral proliferation. Here, the median age in groups of patients affected by either a malignant or a non-malignant disease was similar and the number of TREC was given for unit of blood volume. Moreover, we specifically assessed beta-TREC, a marker of intrathymic proliferation of T-cells undergoing differentiation. Beta-TREC values were also reduced in patients with malignancies, suggesting a mechanistic impact of the disease itself or its treatment on T-cell generation before beta chain TCR recombination, i.e., at the progenitor level and/or after homing to the thymus. In this respect, previous studies indicating the lack of association between TREC and chemotherapy (Petridou et al., 2002; Chen et al., 2005), could argue for a direct impact of hematological malignancy on thymic function rather than for a role of previous treatment.

In conclusion, we have previously shown an association between low levels of sj and beta-TREC and disease recurrence in the group of 27 haplo-HSCT patients with malignancies (Clave et al., 2012). Given the similarity in thymic function recovery of these patients with those treated with UCBT, by adding to the analysis the results obtained for these patients, we could increase to 46 the total number of patient treated for malignancies. With this addition, patients who relapsed still had a lower count of sj and beta TREC (Figures 3A,B) and patients with a low value of beta-TREC, both before and at month 6 after transplantation, had a significantly higher incidence of relapse (Figures 3C,D). Since the cohort size did not allow us to perform a multivariate analysis, we cannot exclude that the effect on relapse could be due to other disease variable. However, none of the different clinical parameters we tested such as disease risk or patient age, had a significant effect on relapse in univariate analysis. Therefore, the significant correlation of beta-TREC with relapse 6 months after transplantation suggest that a post-graft thymic differentiation of naïve T cells with anti-leukemia potential could play a crucial role in preventing relapse and mediating GVL.

### Conflict of interest statement

The authors declare that the research was conducted in the absence of any commercial or financial relationships that could be construed as a potential conflict of interest.
